# The BODECOST Index (BCI): a composite index for assessing the impact of COPD in real life

**DOI:** 10.1186/s40248-016-0045-4

**Published:** 2016-03-03

**Authors:** Roberto W. Dal Negro, Bartolome R. Celli

**Affiliations:** National Centre for Respiratory Pharmacoeconomics and Pharmacoepidemiology, CESFAR, Verona, Italy; Brigham and Women’s Hospital and Harvard Medical School, Boston, MA USA

**Keywords:** BODE index, BODECOST index, COPD, COPD impact, Cost-of-illness, Mortality prediction

## Abstract

**Background:**

Chronic Obstructive Pulmonary Disease (COPD) is a progressive condition which is characterized by a dramatic socio-economic impact.

Several indices were extensively investigated in order to asses the mortality risk in COPD, but the utilization of health care resources was never included in calculations. The aim of this study was to assess the predictive value of annual cost of care on COPD mortality at three years, and to develop a comprehensive index for easy calculation of mortality risk in real life.

**Methods:**

COPD patients were anonymously and automatically selected from the local institutional Data Base. Selection criteria were: COPD diagnosis; both genders; age ≥ 40 years; availability of at least one complete clinical record/year, including history; clinical signs; complete lung function, therapeutic strategy, health BODE index; Charlson Comorbidity Index, and outcomes, collected at the first visit, and over the following 3-years. At the first visit, the health annual cost of care was calculated in each patient for the previous 12 months, and the survival rate was also measured over the following 3 years. The hospitalization and the exacerbation rate were implemented to the BODE index and the novel index thus obtained was called BODECOST index (BCI), ranging from 0 to 10 points. The mean cost for each BCI step was calculated and then compared to the corresponding patients’ survival duration.

Parametrical, non parametrical tests, and linear regression were used; *p* < 0.05 was accepted as the lower limit of significance.

**Results:**

At the first visit, the selected 275 patients were well matched for all variables by gender. The overall mortality over the 3 year survey was 40.4 % (*n* = 111/275). When compared to that of BODE index (*r* = 0.22), the total annual cost of care and the number of exacerbations showed the highest regression value vs the survival time (*r* = 0.58 and *r* = 0.44, respectively). BCI score proved strictly proportional to both the cost of care and the survival time in our sample of COPD patients.

**Discussion:**

BCI takes origin from the implementation of the BODE index with the two main components of the annual cost of care, such as the number of hospitalizations and of exacerbations occurring yearly in COPD patients, and their corresponding economic impact. In other words, higher the BCI score, shorter the survival and higher the cost, these trends being strictly linked.

**Conclusions:**

BCI is a novel composite index which helps in predicting the impact of COPD at 3 years in real life, both in terms of patients’ survival and of COPD economic burden.

## Background

Chronic obstructive pulmonary disease (COPD) is the chronic respiratory condition that currently represents the most significant health problem at international level [1]. The epidemiological, clinical and socio-economic impact of COPD still is constantly increasing, and COPD is projected to be the 3^rd^ leading cause of death in the world by 2030, and the 7^th^ as a burden of disease [2].

Chronic Obstructive Pulmonary Disease (COPD) is a progressive condition which is characterized by a dramatic socio-economic impact [3, 4]. The role of clinical signs, lung function, imaging, biological pictures, and multiple scores (BODE index and other composite indices, such as, BODEx; BODEm; BODEi) was extensively investigated in order to asses the mortality risk in COPD, but with variable results due to their different specificity and sensitivity [5–19].

Even if all these indices have contributed to clarify the different clinical patterns of COPD patients at different risk, the utilization of health care resources was never included, to our knowledge, in the calculation of the mortality risk of these patients.

The aim of this study was to assess the predictive value of COPD annual cost of care on mortality at 3 years, and to develop a comprehensive index for the easy calculation of mortality risk in real life.

## Methods

### The sample

The study sample was anonymously and automatically selected [20] from the local institutional Data Base (period 2008–2012), and selection criteria were: COPD subjects of both genders; age ≥ 40 years; availability of at least one complete clinical record/year, including their history; clinical signs; complete lung function, therapeutic strategy, health BODE index; Charlson Comorbidity Index, and outcomes, collected at the first visit, and over the following 3-years [4].

At the first visit, the health annual cost (direct and indirect cost) was calculated in 275 consecutive COPD patients for the previous 12 months, and their survival was also calculated over the following 3 years.

As the impact is mainly related to the hospitalization and the exacerbation rate per year in these patients, the corresponding values were graded and implemented to the BODE index [6], according to the algorithm reported in Table 1. The novel index thus obtained was called BODECOST index (BCI), ranging from 0 to 10 points and from 1 to 4 severity (such as, 0–2; 3–4; 5–6, and 7–10 points).

**Table 1:** The algorithm for the BODECOST index (BCI)

variables	score			
	0	1	2	3
BODE index	0–2	3–4	5–6	7–10
Hospitalizations (n/y)	0	1	2	>2
Exacerbations (n/y)	0–1	2	>2	

The mean cost for each BCI step was calculated stemming from original data already published in a previous study [4], and then compared to the corresponding patients’ survival (in days) for each BCI step. Finally, the rate of cost by BCI step was also calculated in the whole sample of COPD patients.

### Statistics

Parametrical and non parametrical tests were used for comparing means ± sd; 95 % CI (Confidence Interval) was also calculated. Any relationship between collected variables vs the annual total COPD cost and survival were assessed by linear regression, and *p* < 0.05 was accepted as the lower limit of significance. Calculations for assessing the sensitivity of the novel composite index were based on the data from reference 4.

The present study is a spontaneous research, without any founding, and no conflict of interest to declare.

## Results

The general profile of the whole sample (*n* = 275; males *n* = 226; females *n* = 49) is reported in Table 2 for all variables of the study. At the first visit, patients of both genders were well matched for age, dyspnea, BODE index, lung function, Charlson Comorbiditiy Index (all p = ns). Males had slight, but significant, higher values of FEV_1_ (1.5 L ±0.5sd vs 1.2 L ±0.5sd, respectively; *p* < 0.001) and BMI than females (27.1 ± 4.6sd VS 25.1 ± 4.3sd, respectively; *p* < 0.006), due to their mean size. Males also had a more evident history of smoke (active smokers 36.7 % vs 20.8 %, and never smokers 5.7 % vs 26.6 %, respectively).

**Table 2:** The sample profile

	The whole sample (*n* = 275)
age (y)	70.9 ± 8.4
BMI	26.8 ± 4.6
smoking habit	
active	65 (23.6 %)
ex	184 (66.9 %)
never smoker	26 (9.5 %)
FEV_1_ (I)	1.5 ± .05
FEV_1_ % pred,	55.9 ± 18.8
FEV_1_/FVC (%)	55.9 ± 10.3
FEV_1_/VC	51.1 ± 10.6
RV % pred.	156.9 ± 46.2
TL_CO_/VA % pred	70.9 ± 26.2
FEV_1_ reversibility	6.7 ± 7.9
6’ walking test (mt)	233.3 ± 92.7
MRC score	2.3 ± 1.2
BODE index	4.4 ± 2.6
Charlson comorb. index	3.3 ± 1.8

Patients’ distribution according to GOLD guidelines was: GOLD I = 11.6 %( *n* = 32); GOLD II = 48.4 % (*n* = 133); GOLD III = 32.7 % (*n* = 90), and GOLD IV = 7.3 % (*n* = 20), and it was well matched by gender.

The overall mortality over the 3 year survey was 40.4 % (*n* = 111/275), such as: 91/111 (82.0 % in males (*n* = 91/111), and 18.0 % (20/111) in females, respectively. Mean survival for subjects who deceased over the 3-year period was 676 days instead of 1095 for survivors.

The mean exacerbation rate/patient/year was 1.2 and 1.4 in survivors and in those who died over the 3 years, respectively (+16.7 %). When adjusted for the effective survival duration, the latter switched up to 2.3, and the difference vs that of survivors became substantially higher (+91.7 %).

Correspondingly, the hospitalization rate/patient/year was 0.5 and 0.8 in survivors and in those who died over the 3 years, respectively (+60.0 %). When adjusted for the survival duration, the latter switched up to 1.3, and the difference vs that of survivors highly increased (+160.0 %).

The total annual cost of care and the number of exacerbations showed the highest regression value vs the survival time (*r* = 0.58 and *r* = 0.44, respectively) (Table 3), such as, higher than that of age and that of BODE index.

**Table 3:** Regressions vs survival duration (days)

Age	*p* < 0.001	*r* = − 0.28
BODE	*p* < 0.001	*r* = − 0.21
n. exacerbations	*p* < 0.0001	*r* = − 0.44
n. hospitalizations	*p* < 0.0001	*r* = − 0.32
TOTAL COST	*p* < 0.0001	*r* = − 0.58

Table 4 reports the progression of cost due to hospitalizations + exacerbations, and the survival duration (means ± sd) by the different BCI severity steps. It appears very clear that BCI score proves strictly proportional to both the cost of care and the survival time in our sample of COPD patients, even if it is directly proportional in the former, and inversely proportional in the latter case.

**Table 4:** The relationship between different levels of BCI score, hospitalization + exacerbation cost, and survival (means ± sd)

BCI score	Cost Exac. + Hosp (€) (mean ± sd)	Survival (days) (mean ± sd)
0–2	494.8	1023.8
(*n* = 142)	(1454.2)	(198.9)
3–4	2040.9	889.5
(*n* = 66)	(2079.0)	239.4
5–6	4952.9	762.2
(*n* = 36)	(2265.3)	(283.4)
7–10	9224.9	752.1
(*n* = 31)	(7804.2)	(226.7)

## Discussion

Identifying the risk of mortality in COPD patients still represents a crucial and strategic issue in COPD management. Many aspects had been focused in several studies which used a single physiological measure of lung function (i.e. FEV_1_) [21, 22], rather than complex clinical assessments [8, 12, 23–27], or multidimensional scores [6, 7, 9, 13, 14, 19].

These investigational approaches consented a continuous improvement in the overall definition of COPD mortality risk because several domains had been progressively investigated and valued.

Nevertheless, a wide variability is still persisting, mainly due to the different role played by the variables used in the various studies. FEV_1_ represents the first and the most used variable adopted for predicting COPD mortality [2–22], even if it does not fully mirror the huge complexity of pathogenetic, clinical and functional phenomena related to COPD survival [6].

Also the progression, the prognosis, and consequently the mortality risk in COPD can not be fully explained by the sole FEV_1_ measurement [1].

In general, the introduction of multidimensional grading systems improved the sensitivity of the mortality risk assessment in COPD patients because, even if at different level of specificity, these instruments valued several factors affecting COPD severity and prognosis.

In particular, BODE index (such as, BMI, obstruction, dyspnea, exercise) [6] proved to be the most sensitive and specific from this point of view [9] when compared to other similar multidimensional indices (such as, ADO: age, dyspnea, obstruction; DOSE: dyspnea, obstruction, smoking, exacerbations) [13].

The COPD annual cost of care (or its major components) was never regarded as a feasible contribution in the assessment of mortality risk in this kind of patients to our knowledge, even if the cost of care should likely represent the most comprehensive index which reflects the very final result of all factors affecting COPD severity and progression.

Actually, data from the present study confirmed once again that the BODE index is highly significantly related to the length of survival (*p* < 0.001), even if this relationship was modest (*r* = − 0.21), and a not negligible variability can be presumed when mortality was assessed only by means of the BODE index in our sample of COPD patients.

On the other hand, both the hospitalization and the exacerbation rates seemed more strictly related to the length of survival (such as: *r* = − 0.32 and *r* = − 0.44, respectively), and the corresponding costs showed the same trend. Moreover, the total annual COPD cost of care had the highest value for this relationship (*r* = − 0.58), thus suggesting a much closer correspondence between the annual cost of care and the survival at 3 years in our COPD patients.

The hypothesis concerning the convenience of using the cost of care in predicting COPD mortality at 3 years seems clearly confirmed by the strict correspondence between the progression of the BCI score, the hospitalization/exacerbation cost (which represents more than 70 % of total annual cost), and the duration of survival. In other words, higher the BCI score, shorter the survival and higher the cost, these trends being strictly linked.

Finally, BCI takes origin from the implementation of the BODE index with the two main components of the annual cost of care, such as the number of hospitalizations and of exacerbations occurring yearly in COPD patients. Actually, even if this information can suffer from some variability mainly due to the inaccurate collection of data, the BCI assessment does nor require *per sè* complex measurements, because the implementation of the annual rate of both hospital admissions and exacerbations should be easy to collect and demonstrated by the patient, as well as by the physician who can also easily calculate the corresponding economic impact in his office. BCI definitively maintains the same simplicity of BODE index, even if it provides more sensitive information in terms of the patients’ mortality risk in clinical practice.

The present paper has some weakness points, such as: females only represented the 18 % of the sample. Moreover, both the sensitivity and the specificity of this novel instrument of investigation should be confirmed on bigger population samples.

## Conclusions

BCI is a novel and easy composite index which helps in predicting the impact of COPD at 3 years in real life, both in terms of patients’ survival and of COPD economic burden.

## References

[1] Global Initiative for Chronic Obstructive Lung Disease. Global strategy for the diagnosis, management, and prevention of chronic obstructive pulmonary disease (Updated February 2013), 2013. Available from: http://www.goldcopd.org/Guidelines/guidelines-global-strategy-for-diagnosis-management-2013.html. Accessed 10 December 2014.

[2] Mannino DM, Higuchi K, Yu TC, Zhou H, Li Y, Tian H (2015). Economic burden of chronic obstructive pulmonary disease in the presence of comorbidities. Chest..

[3] Mathers CD, Loncar D (2006). Projections of global mortality and burden of disease from 2002 to 2030. PLoS Med.

[4] Dal Negro RW, Bonadiman L, Turco P, Tognella S, Iannazzo S (2015). Costs of illness analysis in Italian patients with chronic obstructive pulmonary disease (COPD): an update. Clin Econ Outcomes Res..

[5] Traver GA, Cline MG, Burrows B (1979). Predictors of mortality in chronic obstructive pulmonary disease. A 15-year follow-up study. Am Rev Respir Dis.

[6] Celli BR, Cote CG, Marin JM (2004). Casanova C, Montes de Oca M, Mendez RA, et al. The body-mass index, airflow obstruction, dyspnea, and exercise capacity index in chronic obstructive pulmonary disease. N Engl J Med.

[7] Briggs A, Spencer M, Wang H, Mannino D, Sin DD (2008). Development and validation of a prognostic index for health outcomes in chronic obstructive pulmonary disease. Arch Intern Med..

[8] Halpin DM, Peterson S, Larsson TP, Calverly PM (2008). Identifying COPD patients at increased risk of mortality: predictive value of clinical study baseline data. Respir Med..

[9] Celli BR, Cote CG, Lareau SC, Meek PM. Predictors of survival in COPD: more than just FEV1. Respir Med. 2008;102(Suppl1):S27–35. 10.1016/S0954-6111(08)70005-2 18582794

[10] Ko FW, Tam W, Tung AH, Ngai J, Ng SS, Lai K (2011). A longitudinal study of serial BODE indices in predicting mortality and readmissions for COPD. Respir Med..

[11] Gudmundsson G, Ulrik CS, Gislason T, Lindberg E, Brøndum E, Bakke P (2012). Long-term survival in patients hospitalized for chronic obstructive pulmonary disease: a prospective observational study in the Nordic countries. Int J Chron Obstruct Pulmon Dis..

[12] Boutou AK, Shrikrishna D, Tanner RJ, Smith C, Kelly JL, Ward SP (2013). Lung function indices for predicting mortality in COPD. Eur Respir J..

[13] Mortegi T, Jones RC, Ishii T, Hattori K, Kusonoki Y, Furutate R (2013). A comparison of three multidimensional indices of COPD severity as predictors of future exacerbations. Int J Chron Obstruct Pulmon Dis.

[14] Moberg M, Vestbo J, Martinez G, Williams JE, Ladelund S, Lange P (2014). Validation of the i-BODE index as a predictor of hospitalization and mortality in patients with COPD participating in pulmonary rehabilitation. COPD.

[15] Pedone C, Scarlata S, Forastiere F, Bellia V, Antonelli IR (2014). BODE index or geriatric multidimensional assessment for the prediction of very-long-term mortality in elderly patients with chronic obstructive pulmonary disease ? A prospective cohort study. Age Ageing.

[16] Lombolt FK, Laulund AS, Bjarnason NH, Jorgensen HL, Godtfredsen N.S. Meta-analysis of routine blood tests as predictors of mortality in COPD. Eur Clin Respir J. 2014; Jun 5, 1 doi 10.3402/ecrj v1 24110 eCollection 2014 10.3402/ecrj.v1.24110 PMC4629760 26557244

[17] Goossens LMA, Leimer I, Metzdorf N, Becker K, Rutten van Molken MPMH. Does the 2013 GOLD classification improve the ability to predict lung function decline, exacerbation and mortality: a post-hoc analysis of the 4-year UPLIFT trial. BMC Pulm Med. 2014; 14:163. doi: 10.1186/1471-2466-14-163. 10.1186/1471-2466-14-163 PMC4223746 25326750

[18] Chen CZ, Ou CY, Yu CH, Yang SC, Chang HY, Hsiue TR (2015). Comparison of global initiative for chronic obstructive pulmonary disease 2013 classification and body mass index, airflow obstruction, dyspnea, and exacerbations index in predicting mortality and exacerbations in elderly adults with chronic obstructive pulmonary disease. J Am Geriatr Soc..

[19] Navarro A, Costa R, Rodriguez-Carballeira M, Yun S, Lapuerte A, Barrera A (2015). Prognostic assessment of mortality and hospitalizations of outpatients with advanced chronic obstructive pulmonary disease. Usefulness of the CODEX index. Rev Clin Esp.

[20] Boole G (1847). Mathematical analysis of logic, being an essay towards a calculus of deductive reasoning.

[21] Siafakas NM, Vermeire P, Pride NB (1995). Optimal assessment and management of chronic obstructive pulmonary disease (COPD). Eur Respir J..

[22] Pauwels RA, Buist AS, Calverly PM, Jenkins CR, Hurd SS (2001). Global strategy for the diagnosis, management, and prevention of chronic obstructive pulmonary disease: NHLBI/WHO Global initiative for Chronic Obstructive Lung disease (GOLD) Workshop summary. Am J Respir Crit Care Med.

[23] Nocturnal Oxygen Therapy Trial Group (1980). Continuous or nocturnal oxygen therapy in hypoxemic chronic obstructive pulmonary disease: a clinical trial. Ann Int. Med.

[24] Gerardi DA, Lovett L, Benoit-Connors ML, Reardon JZ, ZuWallacK RL (1996). Variables related to increased mortality following outpatient pulmonary rehabilitation. Eur Respir J..

[25] Nishimura K, Izumi T, Tsukino M, Oga T (2002). Dyspnea is a better predictor of 5-year survival than airway obstruction in patients with COPD. Chest..

[26] Schols AM, Slangen J, Volovics L, Wouters EF (1998). Weight loss is reversible factor in the prognosis of chronic obstructive pulmonary disease. Am J Respir Crit Care Med..

[27] Landbo C, Prescott E, Lange P, Vestbo J, Almdal TP (1999). Prognostic value of nutritional status in chronic obstructive pulmonary disease. Am Rev Respir Crit Care Med..

